# Carbonate apatite artificial bone

**DOI:** 10.1080/14686996.2021.1947120

**Published:** 2021-08-16

**Authors:** Kunio Ishikawa, Koichiro Hayashi

**Affiliations:** Department of Biomaterials, Faculty of Dental Science, Kyushu University, Higashi-ku, Japan

**Keywords:** Carbonate apatite, calcium carbonate, dissolution–precipitation reaction granules, honeycomb, coating, 30 Bio-inspired and biomedical materials, 211 Scaffold / Tissue engineering/Drug delivery

## Abstract

Bone apatite is not hydroxyapatite (HAp), it is carbonate apatite (CO_3_Ap), which contains 6–9 mass% carbonate in an apatitic structure. The CO_3_Ap block cannot be fabricated by sintering because of its thermal decomposition at the sintering temperature. Chemically pure (100%) CO_3_Ap artificial bone was recently fabricated through a dissolution–precipitation reaction in an aqueous solution using a precursor, such as a calcium carbonate block. In this paper, methods of fabricating CO_3_Ap artificial bone are reviewed along with their clinical and animal results. CO_3_Ap artificial bone is resorbed by osteoclasts and upregulates the differentiation of osteoblasts. As a result, CO_3_Ap demonstrates much higher osteoconductivity than HAp and is replaced by new bone via bone remodeling. Granular-type CO_3_Ap artificial bone was approved for clinical use in Japan in 2017. Honeycomb-type CO_3_Ap artificial bone is fabricated using an extruder and a CaCO_3_ honeycomb block as a precursor. Honeycomb CO_3_Ap artificial bone allows vertical bone augmentation. A CO_3_Ap-coated titanium plate has also been fabricated using a CaCO_3_-coated titanium plate as a precursor. The adhesive strength is as high as 76.8 MPa, with excellent tissue response and high osteoconductivity.

## Graphical abstract

**Figure:**
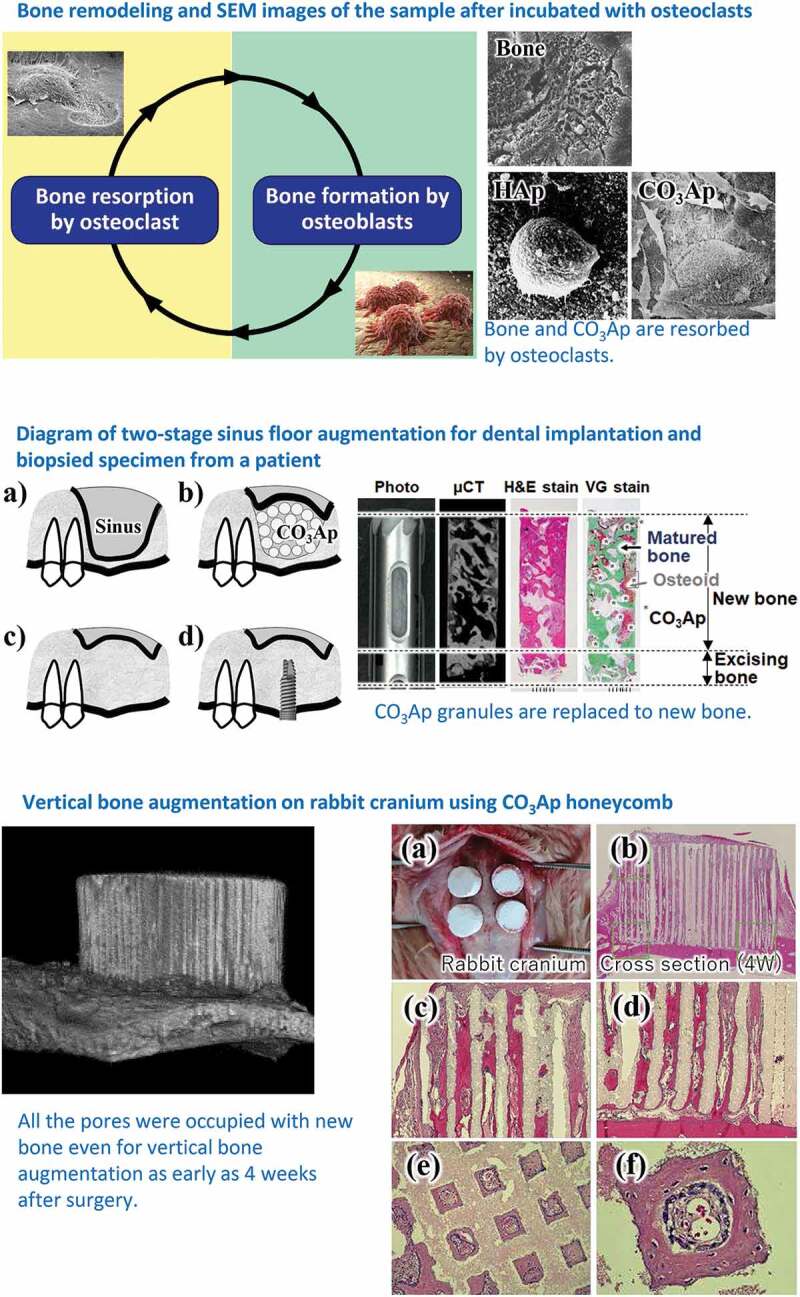

## 1. Bone apatite

Animals with a skeleton are classified as invertebrates or vertebrates. The skeleton of invertebrates is composed of calcium carbonate (CaCO_3_) and is likely derived from elements found in seawater. In contrast, vertebrates, including humans, have a skeleton of carbonate apatite [CO_3_Ap: Ca_10-a_(PO_4_)_6-b_(CO_3_)_c_] instead of CaCO_3_ [1–5].

A key difference between CaCO_3_ and CO_3_Ap skeletons is phosphorous. Phosphorous or phosphate is important in energy metabolism in the process of generating energy or adenosine triphosphate (ATP). Invertebrates can use phosphates present in seawater, even though their concentrations are low (1.3 μmol/L). However, vertebrates living on the land need to store phosphorous in the body. During evolution from invertebrates to vertebrates, bone became the storage organ of phosphorous in vertebrates. In other words, CO_3_Ap was chosen as bone apatite as a result of evolution from invertebrates to vertebrates.

CO_3_Ap or bone apatite should be amenable for use as artificial bone. However, sintered hydroxyapatite (HAp) instead of CO_3_Ap has been used as a typical artificial bone since the 1970s. This is because the thermal decomposition of CO_3_Ap begins at approximately 400°C, which prevents the fabrication of sintered CO_3_Ap. Recently, 100% chemically pure CO_3_Ap blocks were fabricated through a dissolution–precipitation reaction in an aqueous solution using a CaCO_3_ block as a precursor.

## 2. CO_3_Ap fabrication though dissolution–precipitation reaction using a precursor

There are three key requirements for compositional transformation through the dissolution–precipitation reaction. First, the solubility of the precursor should be higher than that of the final product. Second, any component that is lacking must be supplied from the aqueous solution. Third, precipitates or crystals of the final product should have the ability to interlock with one another to maintain the shape of the block.

CO_3_Ap is a thermodynamically stable phase under physiological conditions. It is not soluble under physiological conditions. Moreover, CO_3_Ap crystals can interlock. Many compounds are more soluble than CO_3_Ap, and thus can be precursors for the fabrication of CO_3_Ap through dissolution–precipitation reactions including CaCO_3_ [6–18], α-tricalcium phosphate [19–24], dicalcium phosphate dihydrate [25,26], and CaSO_4_ [27–30]. Moreover, a component that is lacking can be supplied from aqueous solution.

One of the ideal precursors is CaCO_3_. It has low solubility in aqueous solution at neutral pH and contains both calcium and carbonate. Chemically pure CaCO_3_ blocks can be easily fabricated by simply exposing calcium hydroxide [Ca(OH)_2_] compact to carbon dioxide (CO_2_) [eq. (1)].

(1) $$Ca{\left(OH\right)}_{2}+CO_{2}\to CaCO_{3}+H_{2}O$$

The compositional transformation of CO_3_Ap from CaCO_3_ requires phosphate. The CaCO_3_ block needs to be immersed in a phosphate salt solution for this compositional transformation.

When CaCO_3_ is immersed in an aqueous solution, it dissolves and supplies Ca^2+^ and CO_3_^2-^ [eq. (2)]. If other ions are absent, the water becomes saturated with CaCO_3_. However, the situation is different when water contains PO_4_^3−^. In that case, the phosphate salt aqueous solution can be supersaturated with respect to CO_3_Ap when both Ca^2+^ and CO_3_^2-^ are supplied by the dissolution of CaCO_3_. Thus, Ca^2+^, PO_4_^3−,^ and CO_3_^2-^ precipitate as CO_3_Ap [eq (3)], and the precipitated CO_3_Ap crystals interlock with one another. Continuous dissolution–precipitation reactions lead to the compositional transformation from CaCO_3_ to CO_3_Ap, maintaining the macroscopic structure of the precursor.

(2) $$CaCO_{3}\;\to \;Ca^{2+}+C{O_{3}}^{2-}$$

(3) $$Ca^{2+}+P{O_{4}}^{3-}+C{O_{3}}^{2-}\;\to \;Ca_{10-a}{\left(PO_{4}\right)}_{6-b}{\left(CO_{3}\right)}_{c}$$

Other useful precursors are CaSO_4_ · 2H_2_O, α-tricalcium phosphate [α-TCP: Ca_3_(PO_4_)_2_], and dicalcium phosphate dihydrate [DCPD: CaHPO_4_ · 2H_2_O]. However, sulfate ions tend to remain in the apatitic structure when CaSO_4_ · 2H_2_O is used as a precursor. CO_3_Ap containing a small amount of HAp tends to form when α-TCP or DCPD are used as precursors because of the competitive reaction to form CO_3_Ap and HAp. Therefore, CaCO_3_ appears to be an ideal precursor. Fabrication of CO_3_Ap based on compositional transformation through a dissolution–precipitation reaction using CaCO_3_ as a precursor may have arisen during the evolution of vertebrates from invertebrates.

## 3. CO_3_Ap and bone remodeling

### 3.1. Bone remodeling process

**Figure 1.**
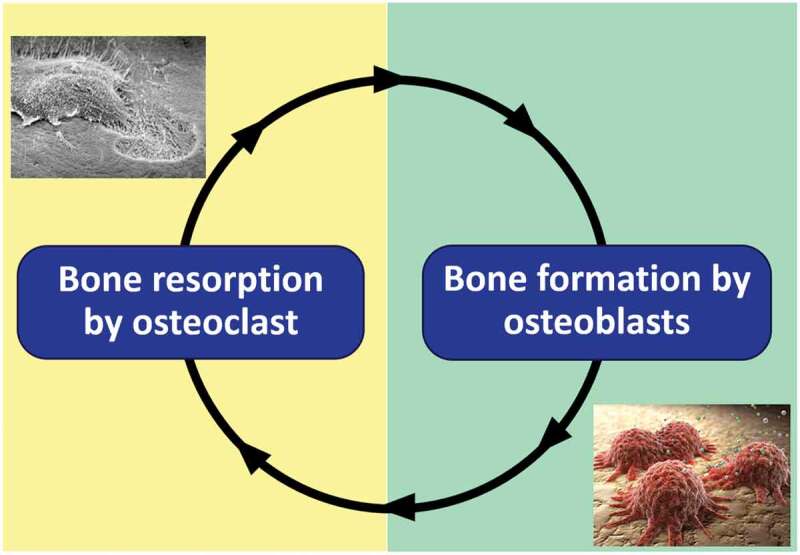
Graphical image of bone remodeling performed by osteoclasts and osteoblasts

**Figure 2.**
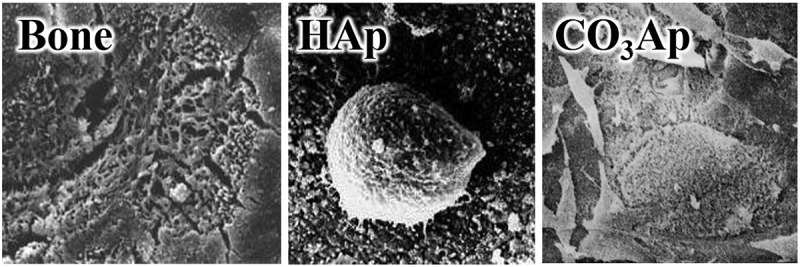
SEM images of bone, sintered HAp, and CO_3_Ap when osteoclasts were incubated on their surfaces [6]

Bone remodeling involves the replacement of old bone and autograft by new bone. Osteoclasts resorb the old bone or autograft, followed by the formation of new bone by osteoblasts (Figure 1). Apatite is osteoconductive. Therefore, osteoblasts are active on their surfaces, although the degree of activity can differ depending on the type of apatite. The activity of osteoclasts also differs according to the type of apatite. Figure 2 displays representative scanning electron microscopy (SEM) images of osteoclasts incubated on the surfaces of bone, HAp, and CO_3_Ap. The absence of osteoclastic resorption with HAp [6] is evidence that HAp cannot be replaced with new bone because osteoclast resorption does not occur. In contrast, osteoclastic resorption occurs for bone and CO_3_Ap.

**Figure 3.**
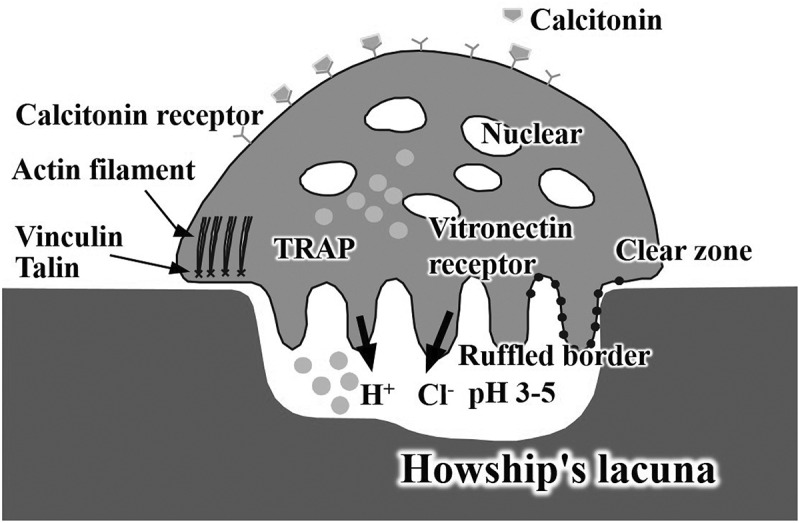
Graphical image of osteoclasts

Osteoclasts form Howship’s lacunae and decrease the pH inside the lacunae to pH 3–5, leading to the dissolution of bone apatite. Thus, osteoclasts resorb apatite by dissolving it using a weak acid (Figure 3).

**Figure 4.**
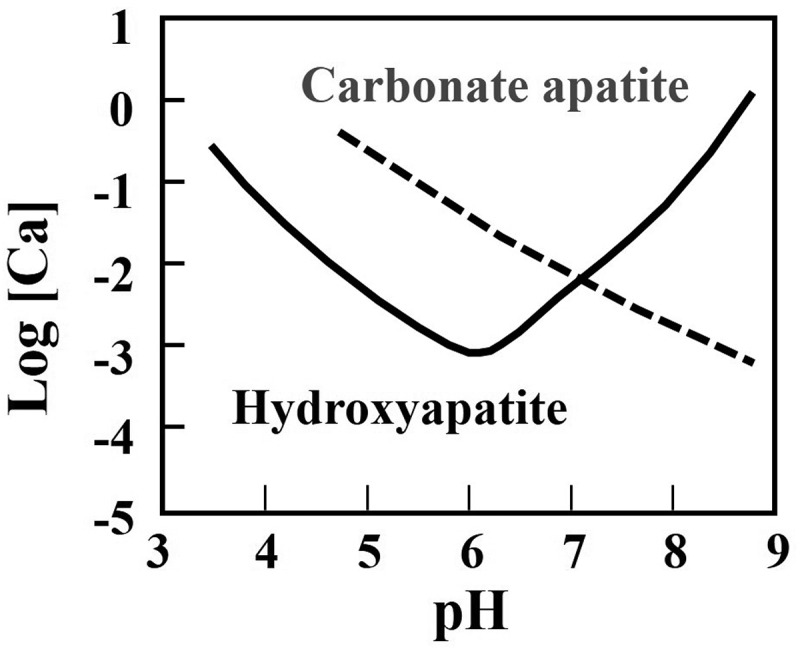
Solubility of carbonate apatite and hydroxyapatite in body fluid in terms of calcium concentration as a function of pH [14]

Figure 4 summarizes the solubilities of HAp and CO_3_Ap as a function of pH. At physiological pH or pH 7.4, CO_3_Ap is thermodynamically the most stable phase. This may explain why bone apatite is CO_3_Ap. However, under weakly acidic conditions produced by osteoclasts or at a pH of 3–5, the solubility of CO_3_Ap is higher than that of HAp. Therefore, CO_3_Ap dissolves in weakly acidic conditions and is resorbed by osteoclasts, whereas HAp does not appreciably dissolve under weakly acidic conditions and is not resorbed by osteoclasts.

### 3.2. Differentiation of osteoblasts

**Figure 5.**
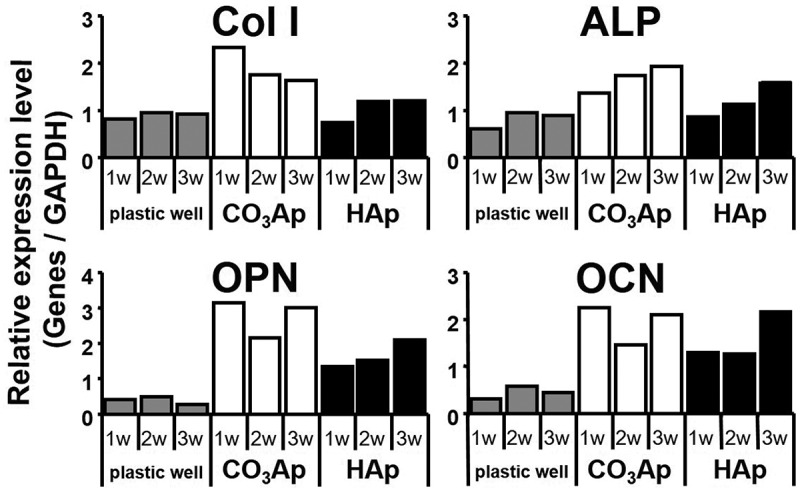
Relative gene expression levels of type I collagen, alkaline phosphatase, osteopontin, and osteocalcin on the surface of plastic well, CO_3_Ap, and sintered HAp [31]

Osteoblastic activity is the counterpart of osteoclastic activity in bone remodeling (Figure 3). One of the parameters of osteoblastic activity is differentiation. Osteoblastic differentiation markers include type I collagen, alkaline phosphatase, osteopontin, and osteocalcin (Figure 5) [31]. Human bone marrow cells (hBMCs) incubated on CO_3_Ap demonstrated much higher expression than HAp. Upregulation of osteoblast differentiation is likely one of the causes of the higher osteoconductivity of CO_3_Ap artificial bone, in addition to the activation of osteoblasts through cell-cell interactions with osteoclasts.

### 3.3. Histological findings

**Figure 6.**
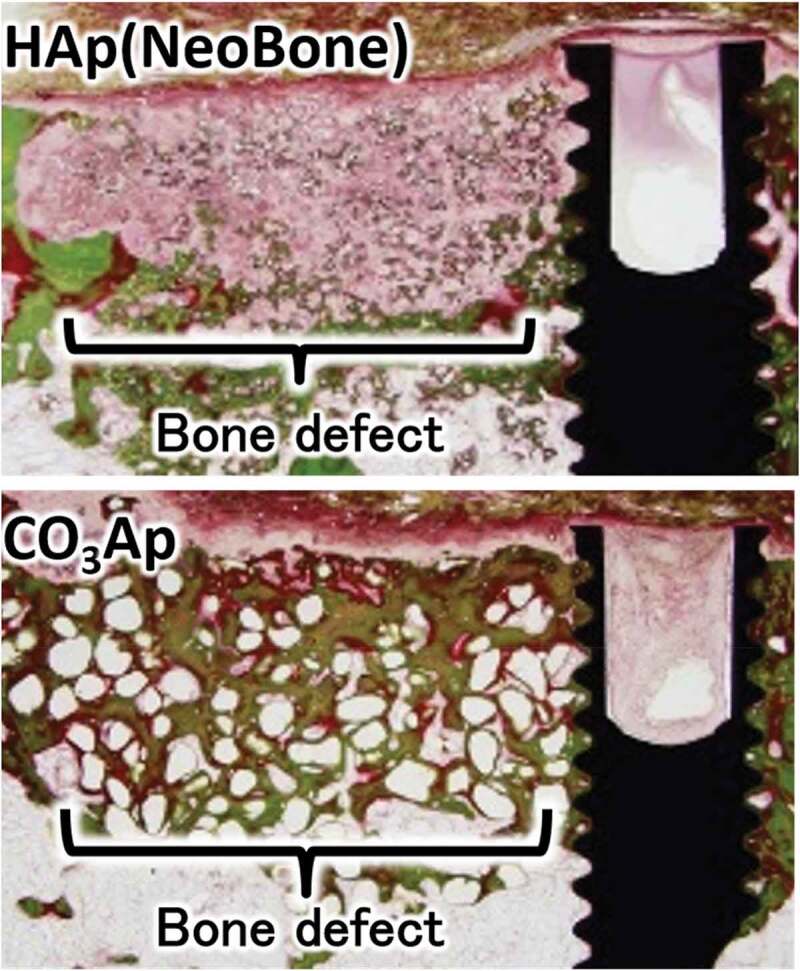
VG-stained histological image of sintered HAp (Neobone®) and CO_3_Ap 4 weeks after reconstruction surgery of beagle dogs’ mandibular bone defect adjacent to a dental implant [32]

Figure 6 summarizes the representative results of Villanueva Goldner (VG) histologic staining comparison of CO_3_Ap and sintered HAp (Neobone®) used for reconstruction of mandibular bone defects adjacent to dental implants in beagle dogs 4 weeks after reconstruction surgery [32]. In VG staining, mature bone is stained green. Reconstruction of a defect with HAp involves the formation of only a limited amount of bone at the defect site, on the surface of the bone defect, and adjacent to the dental implant. This is one reason why no artificial bones have been approved for implant-related bone defect reconstruction surgeries in Japan. In contrast, more bone forms even at the center of bone defects reconstructed using CO_3_Ap. The surfaces of bone defects and dental implants become covered with bone. The documented bonding that results between bone, CO_3_Ap, and dental implant clearly indicating the usefulness of CO_3_Ap as an artificial bone for dental implants.

### 3.4. Clinical trials

**Figure 7.**
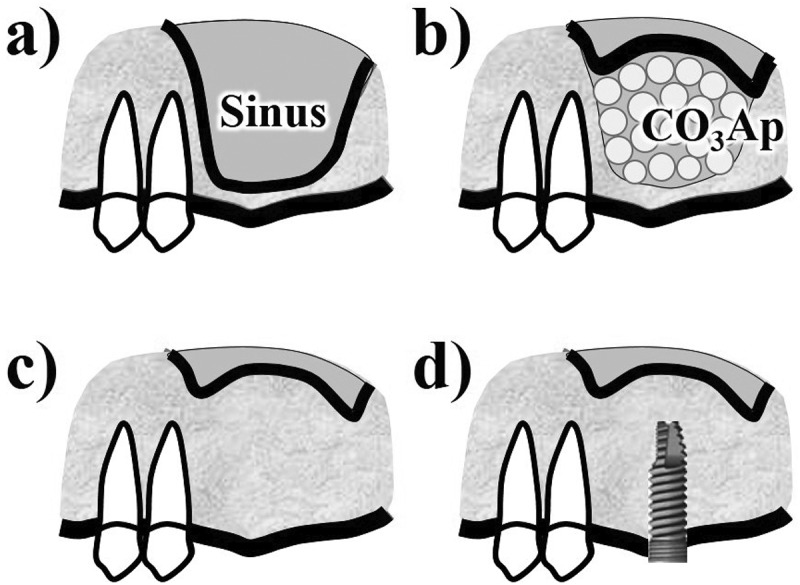
Diagram of two-stage sinus floor augmentation for dental implantation. (a) before operation, (b) filling CO_3_Ap granule after elevation of sinus floor membrane, (c) before biopsy which was performed 8 ± 2 months after the surgery, (d) after dental implantation

The first human clinical trial was performed at three university hospitals in patients requiring sinus floor augmentation [33,34]. Figure 7 illustrates the procedure for two-stage sinus floor augmentation [34]. After the sinus floor membrane was elevated with a mucosal elevator, CO_3_Ap granules were placed in the elevated space (Figure 7(b)). Implant placement was planned for 8 ± 2 months after the augmentation. Prior to implantation, a bone biopsy can be performed using a trephine bur (Figure 7(c)).

**Figure 8.**
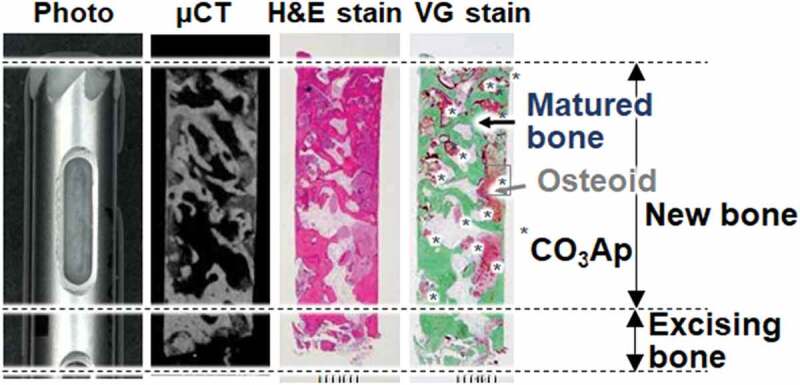
Photo, μ-CT scanning, H-E and VG staining of a histological specimen biopsied from a patient who underwent two-stage sinus floor augmentation for dental implantation [34]

Figure 8 summarizes the micro-computed tomography (μ-CT) images and the appearance of biopsy tissue stained with hematoxylin and eosin (H-E) or VG. CO_3_Ap granules were replaced with new bone, even though a small amount of CO_3_Ap granules remain at this stage [34]. The presence of both mature bone and osteoids indicated active bone remodeling. Few inflammatory cells or foreign-body giant cells were observed in the biopsy specimens. The mean preoperative residual bone height of 3.4 ± 1.3 mm was increased to 13.0 ± 1.9 mm by the sinus floor augmentation using the CO_3_Ap granules. Since the height after sinus floor augmentation is sufficient for dental implants, all patients received dental implants without any problems [33,34]. Based on the trial results, CO_3_Ap granules were approved for clinical use as Cytrans Granules (GC Co, Tokyo, Japan) by the Pharmaceuticals and Medical Devices Agency (PMDA) in 2017. The chemically pure CO_3_Ap granules are the first to be commercially available globally and the first artificial bone that can be used for bone reconstruction aimed at dental implantation in Japan.

## 4. CO_3_Ap honeycomb artificial bone

**Figure 9.**
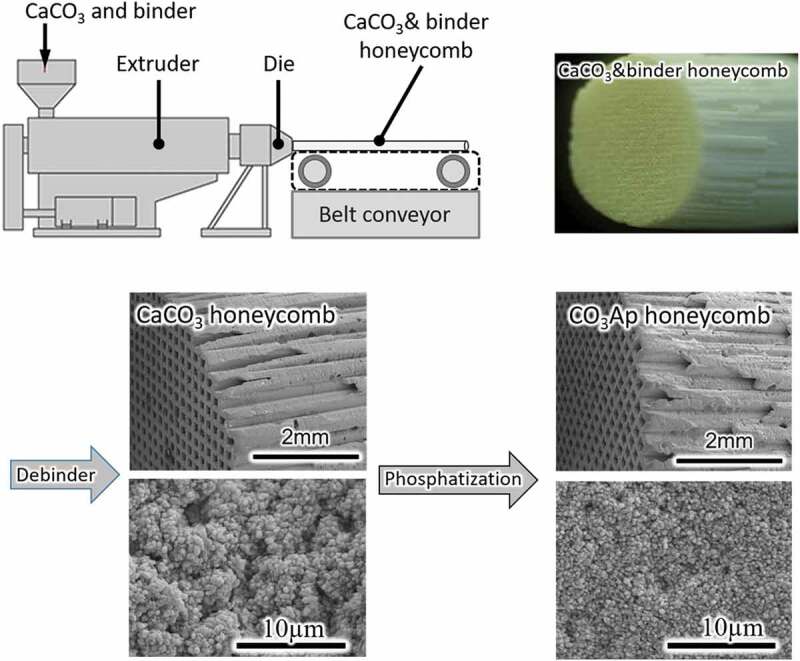
Diagram of honeycomb fabrication process and SEM images of CaCO_3_ and CO_3_Ap honeycombs

Not only composition but also architecture play important roles in governing the ability of artificial bone. In other words, regulation of architecture may be one of the important keys for artificial bone to demonstrate the similar osteoconductivity of autograft. One of the attractive architectures is the honeycomb. Fabrication of the CO_3_Ap honeycomb by the dissolution–precipitation reaction requires a precursor. In general, a honeycomb is fabricated by extruding a raw material through a honeycomb die (Figure 9). An organic binder is necessary for extrusion. Therefore, the organic binder must be eliminated in the subsequent debindering.

The CaCO_3_ honeycomb becomes a CO_3_Ap honeycomb by immersion in a phosphate salt aqueous solution based on compositional transformation through a dissolution–precipitation reaction that maintains the honeycomb structure. Figure 9 shows typical SEM images of CaCO_3_ and CO_3_Ap honeycombs. The macroscopic honeycomb structure was maintained during the compositional transformation. However, the microstructure is changed during the dissolution–precipitation reaction, indicating a dependence on the crystal structure of each honeycomb composition.

**Figure 10.**
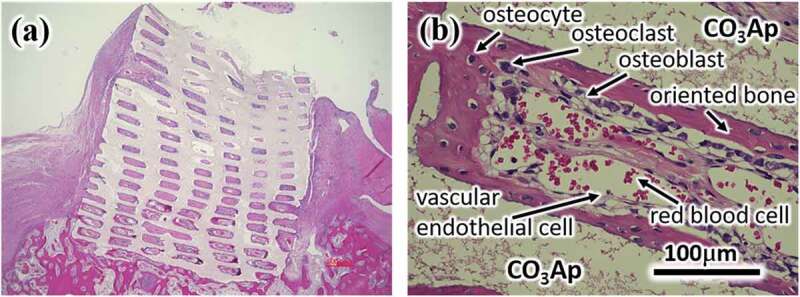
H-E stained histological image 4 weeks after rabbit femoral bone defect was reconstructed using a CO_3_Ap honeycomb [13]. The (b) is a locally magnified photograph of (a)

Figure 10 displays a representative histological image one month after reconstruction of the femoral bone defect using the CO_3_Ap honeycomb [13]. Tissue penetration into all pores of CO_3_Ap honeycomb is evident. At higher magnification, the new bone formation at the pore surface of CO_3_Ap honeycomb is evident. The presence of osteoclasts and osteoblasts on the surface of the newly formed bone indicates active bone remodeling. Osteocytes are also observed within the bone matrix. Interestingly, numerous vascular endothelial cells and red blood cells are found inside the pores, indicating the formation of blood vessels [12].

**Figure 11.**
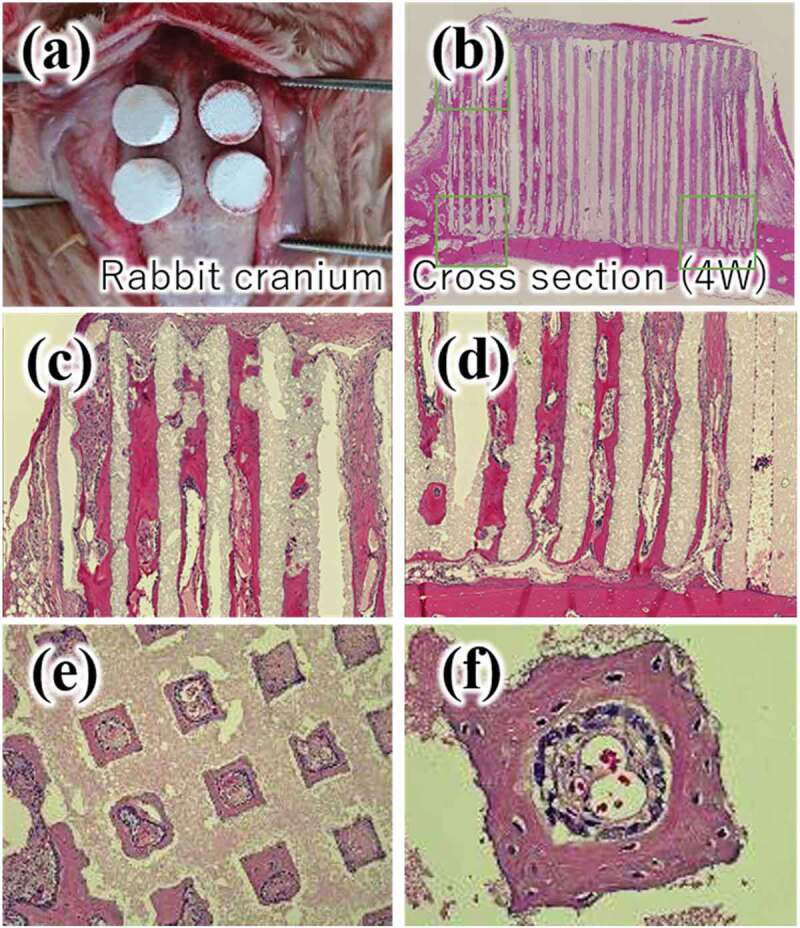
Implantation procedure (a) and H-E stained histological images, (b)-(f), 4 weeks after vertical bone augmentation on rabbit cranium [13]. (c) top of the CO_3_Ap honeycomb, (d) bottom parts of the CO_3_Ap honeycomb, (e) CO_3_Ap honeycomb at mid-height, (f) higher magnification of (e)

Vertical bone augmentation can be performed using the CO_3_Ap honeycomb. Figure 11 summarizes the results of vertical bone augmentation on rabbit cranium. New bone formation was confirmed 4 weeks after implantation, even at the top of the CO_3_Ap honeycomb (Figure 11(b)). Magnified images of the top (Figure 11(c)) and bottom (Figure 11(d)) parts of the CO_3_Ap honeycomb along the pores, cross section of the CO_3_Ap honeycomb at mid-height (Figure 11(e)), and new bone formation at the pore surface of the CO_3_Ap honeycomb (Figure 11(f)) are shown. The same histological results were obtained for vertical bone augmentation as for femoral bone defect augmentation. Osteoclasts, osteoblasts, and osteocytes were also observed. In addition, numerous vascular endothelial cells and red blood cells were found inside the pores.

**Figure 12.**
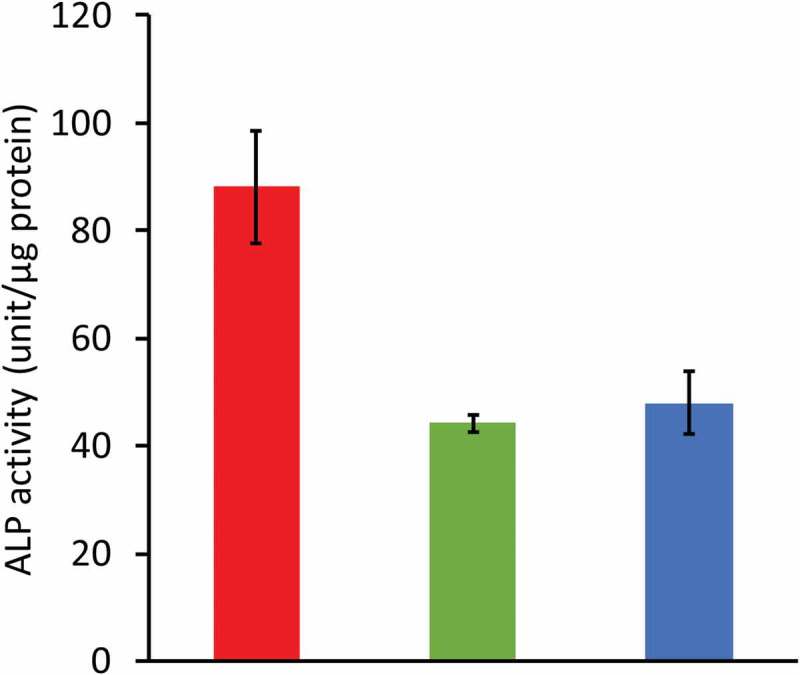
Effects of honeycomb’s composition on MC3T3-E1 cell differentiation *in vitro*. ALP activity of cells cultured for 7 days on CO_3_Ap, HAp, and β-TCP honeycombs [35]

A comparison of the cell and tissue responses among the CO_3_Ap, HAp, and β-TCP honeycombs has also been reported [35]. Alkaline phosphatase (ALP) activity is an index of osteoblast maturation. ALP activity of MC3T3-E1 cells 7 days following seeding was approximately two-fold higher for the CO_3_Ap honeycomb than for β-TCP and HAp honeycombs (Figure 12).

**Figure 13.**
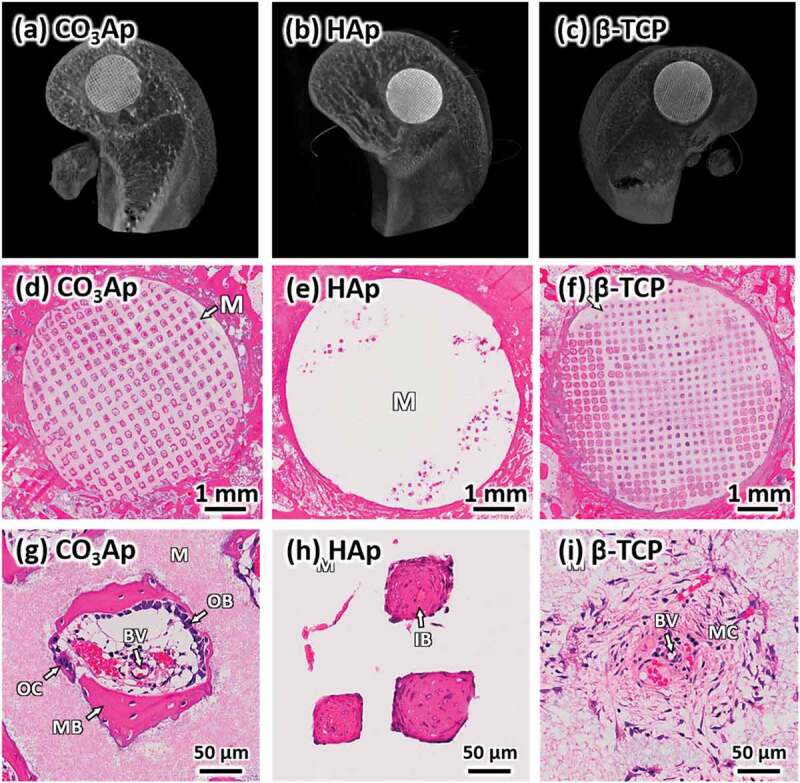
μ-CT images, (a–c), and HE-stained histological images, (d-i), of bone defects 4 weeks after reconstruction with CO_3_Ap (a, d, g), HAp (b, e, h), and β-TCP (c, f, i) honeycombs. ‘OB,’ ‘OC,’ ‘MB,’ ‘IB,’ ‘MC,’ ‘M,’ ‘BV,’ and ‘AC’ indicate osteoblast, osteoclast, new mature bone, immature bone, mesenchyme, material, blood vessel, and adipose cell, respectively [35]

**Figure 14.**
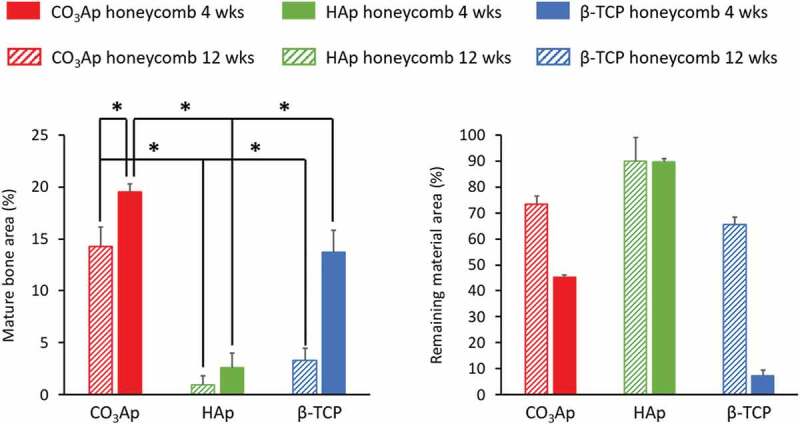
Mature bone area and remaining material area of bone defects 4 and 12 weeks after reconstruction with CO_3_Ap, HAp, and β-TCP honeycombs. *p < 0.05 [35]

Figure 13 summarizes μ-CT and H-E stain histological findings 4 weeks after bone defects made at the distal epiphysis of rabbit femurs were reconstructed with CO_3_Ap, HAp, and β-TCP honeycombs [35]. In the case of the CO_3_Ap honeycomb, new mature bone formed along the walls surrounding the macropores. Osteoblasts were detected along the new bone, osteoclasts were detected on the material surface, and blood vessels were detected in the macropores (Figure 13(c),Figure 13(g)). In the HAp honeycomb, immature bone, but not mature bone, was observed in only a small portion of the macropores at 4 weeks post-operation (Figure 13(e,Figure 13(h)). Osteoblasts, osteoclasts, and blood vessels were not observed in any macropores in the HAp honeycomb, whereas these cells and tissues were present in every macropore examined in the CO_3_Ap honeycomb. Even at 12 weeks after surgery, almost all macropores were occupied by immature bone, and osteoblasts and osteoclasts were not observed in the macropores. In the case of β-TCP honeycomb 4 weeks after surgery, almost all macropores were filled with mesenchyme, and no osteoblasts or osteoclasts were observed in the macropores (Figure 13(f,Figure 13(i)). New mature bone formation was observed within a small portion of macropores. The area of mature bone at 4 and 12 weeks after surgery is summarized in Figure 14 along with the remaining mineral area. The area of mature bone area was significantly larger for CO_3_Ap honeycomb compared to that of HAp and β-TCP honeycombs. Remaining materials area was largest for HAp honeycomb followed by CO_3_Ap honeycomb and β-TCP honeycomb for both 4 and 12 weeks after surgery.

## 5. CO_3_Ap coated titanium plate

Titanium (Ti) is used as an implant device because of its high mechanical strength and ease of osseointegration [36,37]. In particular, surface-roughed Ti implants demonstrate greater osseointegration and a higher implantation success rate. However, osteogenesis of Ti, even with a roughened surface, is poorer than that of osteoconductive materials like apatite. Therefore, initial loosening of Ti implants is a problem for early loading [38, 39, 40].

Ti roughened surface coated with CO_3_Ap may be an ideal Ti implant because of its pronounced osteoconductivity and replacement by new bone. CO_3_Ap coated Ti (CO_3_Ap–Ti) can be fabricated by compositional transformation through a dissolution–precipitation reaction using calcite-coated titanium (CaCO_3_–Ti) as a precursor [39–41].

In this process, CaCO_3_–Ti is established on the roughened surface Ti by wetting the Ti with an ethanol solution of Ca(NO_3_)_2_, followed by heating at 550°C in a CO_2_ gas atmosphere. This results in the thermal decomposition of Ca(NO_3_)_2_ and its carbonation. CO_3_Ap–Ti is then fabricated through a dissolution–precipitation reaction in an aqueous solution of Na_2_HPO_4_ using CaCO_3_–Ti as a precursor.

**Figure 15.**
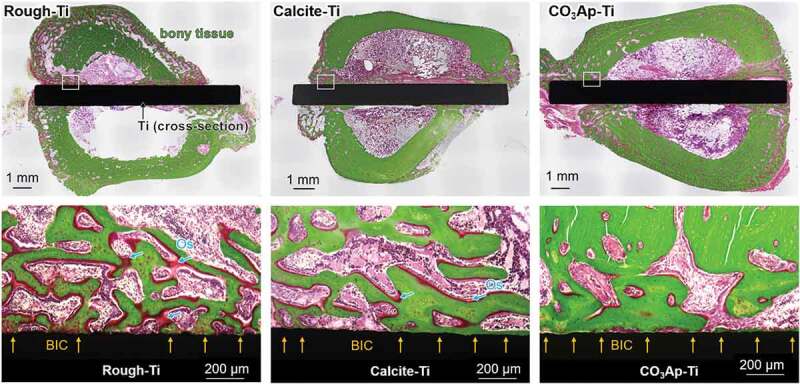
Villanueva-Goldner-stained histological sections: general view, and high-magnification view of the cortical bone of sections in rough-Ti, calcite-Ti, and CO_3_Ap-Ti after 4 weeks of healing showing bone-implant contact (BIC) and osteoid (Os). Yellow arrows indicate BIC areas [41]

The adhesion strengths of CaCO_3_–Ti and CO_3_Ap–Ti are as high as 56.6 and 76.8 MPa, respectively. Figure 15 summarizes the VG-stained histological results 4 weeks after implantation in the straight defect of the proximal tibia 2 cm away from the knee joint in rabbits. Abundant mineralized bone formed on the surface of CO_3_Ap–Ti. In contrast, osteoid, rather than mineralized bone, was primarily present on the surface of the rough–Ti surface. The bone-implant contact percentages in CO_3_Ap–Ti to bone (72.9% ± 6.4%) exceeded those of rough–Ti to bone (48.2 ± 7.8%) and calcite–Ti to bone (59.9 ± 15.4%).

**Figure 16.**
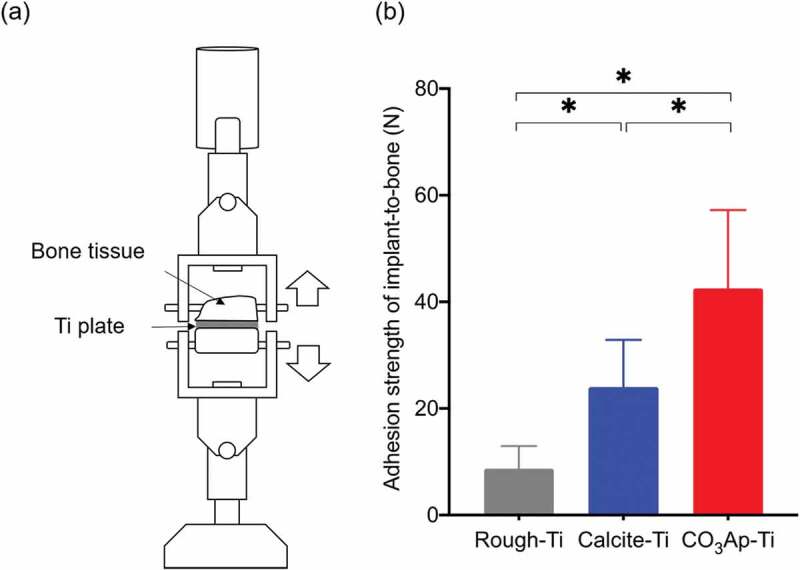
Illustration of the (a) detaching test in bone harvested at 4 weeks after implantation of Ti plates in the tibia defects of rabbits, and (b) adhesion strength of bone-to-implant in rough-Ti, calcite-Ti, and CO_3_Ap-Ti (n = 8). *p < 0.05 for comparisons between the indicated groups [41]

Figure 16 illustrates the detaching test used to measure the adhesion strength of the implant to the bone and the results. The increased bone-implant contact resulted in markedly greater adhesion strengths of CO_3_Ap–Ti to bone (42.5 ± 14.7 N) than that of rough-Ti to bone (8.7 ± 4.3 N) and calcite–Ti to bone (24.0 ± 8.9 N). Most of the fracture occurred between at the bone in the case of CO_3_Ap–Ti, whereas most of the fracture occurred at the interface between Ti plate and calcite for calcite–Ti, and interface between Ti plate and bone for rough-Ti. Very high bonding strength with bone and fracture at bone may be caused, at least in part, to the expansion of CO_3_Ap in the roughened Ti during the dissolution–precipitation reaction.

The documented excellent tissue response, higher osteoconductivity, and higher adhesion strength guarantee the usefulness of CO_3_Ap–Ti for dental and orthopedic implants.

## 6. Conclusion

Chemically pure (100%) CO_3_Ap artificial bone can be fabricated by compositional transformation through a dissolution–precipitation reaction using a precursor. Although the results obtained to date are encouraging, little is known about CO_3_Ap artificial bone compared with other artificial bones, such as HAp and β-TCP.
